# Chromosomes of Asian cyprinid fishes: Variable karyotype patterns and evolutionary trends in the genus *Osteochilus* (Cyprinidae, Labeoninae, “Osteochilini”)

**DOI:** 10.1590/1678-4685-GMB-2020-0195

**Published:** 2020-11-06

**Authors:** Pasakorn Saenjundaeng, Weerayuth Supiwong, Francisco M. C. Sassi, Luiz A. C. Bertollo, Petr Rab, Rafael Kretschmer, Alongklod Tanomtong, Chatmongkon Suwannapoom, Montri Reungsing, Marcelo de Bello Cioffi

**Affiliations:** 1 Faculty of Interdisciplinary Studies, Khon Kaen University, Nong Khai Campus, Nong Khai 43000, Thailand.; 2 Universidade Federal de São Carlos, Departamento de Genética e Evolução, São Carlos, SP, Brazil.; 3 Czech Academy of Sciences, Institute of Animal Physiology and Genetics, Laboratory of Fish Genetics, Rumburská 89, Libechov 277 21, Czech Republic.; 4 Universidade Federal do Rio Grande do Sul, Programa de Pós-Graduação em Genética e Biologia Molecular, Porto Alegre, RS, Brazil.; 5 KhonKaen University, Faculty of Science, Department of Biology, Muang, KhonKaen 40002, Thailand.; 6 University of Phayao, School of Agriculture and Natural Resources, Department of Fishery, Muang, Phayao 56000, Thailand.; 7 Rajamangala University of Technology Tawan-ok, Faculty of Science and Technology, Department of Biotechnology, Siracha, Chonburi 20110, Thailand.

**Keywords:** Fish cytogenetics, karyotype evolution, repetitive DNAs, Thai ichthyofauna

## Abstract

The Cyprinidae family is a highly diversified but demonstrably monophyletic lineage of cypriniform fishes. Among them, the genus *Osteochilus* contains 35 recognized valid species distributed from India, throughout Myanmar, Laos, Thailand, Malaysia, Indonesian archipelago to southern China. In this study, karyotypes and other chromosomal characteristics of five *Osteochilus* species occurring in Thailand, namely *O. lini*, *O. melanopleura*, *O. microcephalus*, *O. vittatus* and *O. waandersii* were examined using conventional and molecular cytogenetic protocols. Our results showed they possessed diploid chromosome number (2n) invariably 2n = 50, but the ratio of uni- and bi-armed chromosomes was highly variable among their karyotypes, indicating extensive chromosomal rearrangements. Only one chromosome pair bearing 5S rDNA sites occurred in most species, except *O. melanopleura*, where two sites were detected. In contrast, only one chromosomal pair bearing 18S rDNA sites were observed among their karyotypes, but in different positions. These cytogenetic patterns indicated that the cytogenomic divergence patterns of these *Osteochilus* species were largely corresponding to the inferred phylogenetic tree. Similarly, different patterns of the distributions of rDNAs and microsatellites across genomes of examined species as well as their different karyotype structures indicated significant evolutionary differentiation of *Osteochilus* genomes.

## Introduction

The Cyprinidae family (sensu Tan and Ambruster, 2018), i.e. *sensu stricto*, is now restricted to phylogenetically and taxonomically highly diversified but a demonstrably monophyletic lineage of cypriniform fishes (Yang *et al.*, 2015) which itself encompasses eleven intra-clade monophyletic lineages taxonomically recently recognized as subfamilies by Tan and Ambruster (2018). One of these lineages, Labeoninae, was demonstrated as sister basal lineage of all remaining cyprinid subfamilies (Conway, 2011; Yang *et al.*, 2015; Stout *et al.*, 2016). Moreover, the lineage monophyly of labeonine cyprinids was supported by both morphological and molecular studies (see review by Yang *et al.*, 2012). These authors also identified four monophyletic intra-lineage groups within Labeoninae, taxonomically recognized (Tan and Ambruster, 2018) as tribes Garrini, Labeonini and taxonomically informal “Osteochilini” and “Semilabeonini”: Labeonine cyprinids are highly morphologically diversified and include altogether around 50 genera with more than 500 species (Eschmeyer Catalog of Fishes, 2020), “Osteochilini” itself contains eight genera with close to 100 recently recognized species.

The genus *Osteochilus* (Günther, 1868) contains 35 recognized valid species distributed from India, throughout Myanmar, Laos, Thailand, Malaysia, Indonesian archipelago to southern China (Karnasuta, 1993). Although three major systematic revisions have been performed for this genus (Karnasuta, 1993), just eight species were included in detailed molecular phylogenetic analyses performed by Yang *et al.* (2012). Although the cytogenetic analysis are restricted, up to now, to only three species, the results point for a quite large karyotype differentiation inside *Osteochilus* (Table 1). 

**Table 1 -:** Available cytogenetic data for *Osteochilus* species.

Species	2n	Karyotype	References
*Osteochilus hasselti*	2n = 50	30m+14sm+6st	Magtoon and Arai, 1990
*O. vittatus*	2n = 50	16m+30sm+4st	Magtoon and Arai, 1990
*O. waandersi*	2n = 50	18m+24sm+4st+4a	Magtoonand Arai, 1993

Cypriniform cytotaxonomy documents a great 2n variation, ranging from 42 in *Acheilognathus gracilis* (Acheilognathidae) (Hong and Zhou, 1985) to 446 in *Diptychus dipogon* (Cyprinidae) (Yu and Yu, 1990). However, 2n = 50 is the most frequent chromosome number, which represents a basal pattern for the whole group (Wolf *et al.*, 1969; Sola and Gornung, 2001). Moreover, several polyploidization events have taken an important role in 2n variation for cyprinids and differentiated sex chromosomes seem rare (Buth *et al.*, 1991; Rab and Collares-Pereira, 1995; Yang *et al.*, 2015).

This study aimed to analyze karyotypes and other chromosomal characteristics as revealed by conventional (Giemsa-staining and C-banding) and molecular (rDNA and microsatellite FISH) protocols in five species of the genus *Osteocheilus* occurring in Thailand, namely *O. lini*, *O. melanoptera*, *O. microcephalus*, *O. vittatus,* and *O. waandersii* together with a brief overlook of cytotaxonomy of “osteochiline” cyprinids. The results added new informative characters useful in comparative genomics at the chromosomal level and highlighted extensive diversity among the analyzed species.

## Material and Methods

### Individuals, mitotic chromosome preparation and C-banding

**Figure 1 -:**
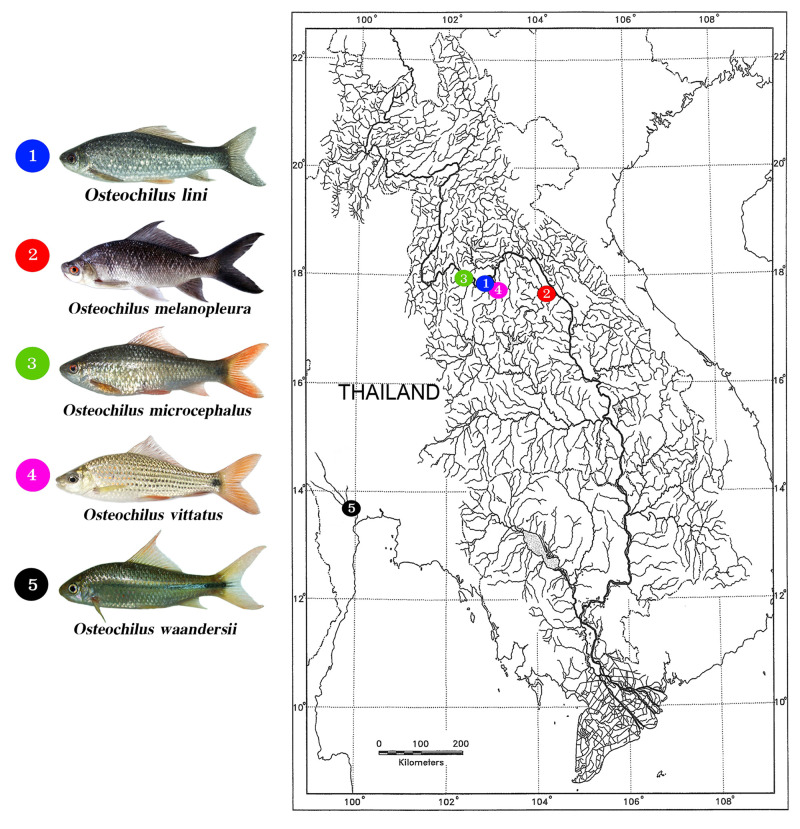
Thailand map showing the collection sites of the five species studied. 1. *Osteochilus lini* (blue circles); 2. *Osteochilus melanopleura* (red circles); 3. *Osteochilus microcephalus* (green circles)*;* 4. *Osteochilus vittatus* (pink circles); 5. *Osteochilus waandersii* (black circles). The maps were created using the following softwares: QGis 3.4.3, Inkscape 0.92 and Photoshop 7.0.

Representatives of five *Osteochilus* species were collected from distinct natural ecosystems of wild regions in Thailand (Figure 1). The numbers and sexes of the individuals under study were presented in Table 2. The specimens were deposited in the fish collections of the Cytogenetic Laboratory, Department of Biology, Faculty of Science (KhonKaen University). Mitotic chromosomes were obtained from anterior kidney, by the conventional air-drying method (Bertollo *et al.*, 2015). The distribution of C-positive heterochromatin blocks was visualized according to Sumner (1972). All the experiments followed ethical protocols, and anesthesia was conducted with clove oil before the sacrifice of the animals. The process was approved by the Animal Ethics Committee of KhonKaen University based on the Ethics of Animal Experimentation of the National Research Council of Thailand AEKKU23/2558. 

**Table 2 -:** Species analyzed, collection sites and number of analyzed individuals (n).

Species	Locality	n
1. *Osteochilus lini*	Mekong Basin	04♀; 05♂
	17°48’48.7”N 102°43’43.8”E	
	Kom KoSubdistrict, Mueang Nong Khai District, Nong Khai	
2. *Osteochilus melanopheura*	Mekong Basin	06♀; 06♂
	17°39’33.3”N 104°16’32.2”E	
	Si SongkhramSubdistrict, Si Songkhram District, Nakhon Phanom	
3. *Osteochilus microcephalus*	Mekong Basin	06♀; 07♂
	17°51’06.5”N 102°35’15.3”E	
	Kong NangSubdistrict, Tha Bo District, Nong Khai	
4. *Osteochilus vittatus*	Mekong Basin	12♀; 10♂
	17°49’06.0”N 102°44’09.8”E	
	Kom KoSubdistrict, Mueang Nong Khai District, Nong Khai	
5. *Osteochilus waandersii*	Mae Klong Basin	04♀; 04♂
	13°47’20.2”N 99°51’44.1”E	
	Nakhon ChumSubdistrict, Ban Pong District, Ratchaburi	

Sites 1 to 5 correspond to the localization of each collection region shown in Figure 1.

### Fluorescence *in situ* hybridization (FISH)

Fluorescence *in situ* hybridization experiments were performed under high stringency conditions (Yano *et al.*, 2017) to identify both classes of ribosomal DNA and microsatellites (CA)_15_, (GA)_15_, (GC)_15_, (A)_30_, (CAC)_10_ and (CGG)_10_ sequences. Two tandemly-arrayed DNA sequences isolated from the genome of *Hoplias malabaricus*, previously cloned into plasmid vectors and propagated in competent cells of *Escherichia coli* DH5α (Invitrogen, San Diego, CA, USA), were used. The first probe contained a 5S rDNA repeat copy and included 120 base pairs (bp) of the 5S rRNA transcribing gene and 200 bp of the non-transcribed spacer (NTS) (Martins *et al.*, 2006). The second probe corresponded to the 1400 bp segment of the 18S rRNA gene obtained via PCR from the nuclear DNA (Cioffi *et al.*, 2009). Both probes were directly labeled with the Nick-Translation mix kit (Roche, Manheim, Germany). The 5S rDNA was labeled with Spectrum Orange-dUTP, and the 18S rDNA was labeled with Spectrum Green-dUTP(Vysis, Downers Grove, IL, USA), according to the manufacturer’s manual. The microsatellite sequences were directly labeled with Cy-3 during the synthesis, as described by Kubat *et al.* (2008).

### Karyotyping and image processing

To confirm the 2n and the results of hybridization experiments, at least 30 metaphase spreads were analyzed per individual. Images were captured with an Axioplan II microscope (Carl Zeiss Jena GmbH, Germany) with CoolSNAP, and processed using an Image-Pro Plus 4.1 software (Media Cybernetics, Silver Spring, MD, USA). Chromosomes were classified according to their arm ratios as metacentric (m), submetacentric (sm), subtelocentric (st), and acrocentric (a) (Levan *et al.*, 1964).

## Results

**Figure 2 -:**
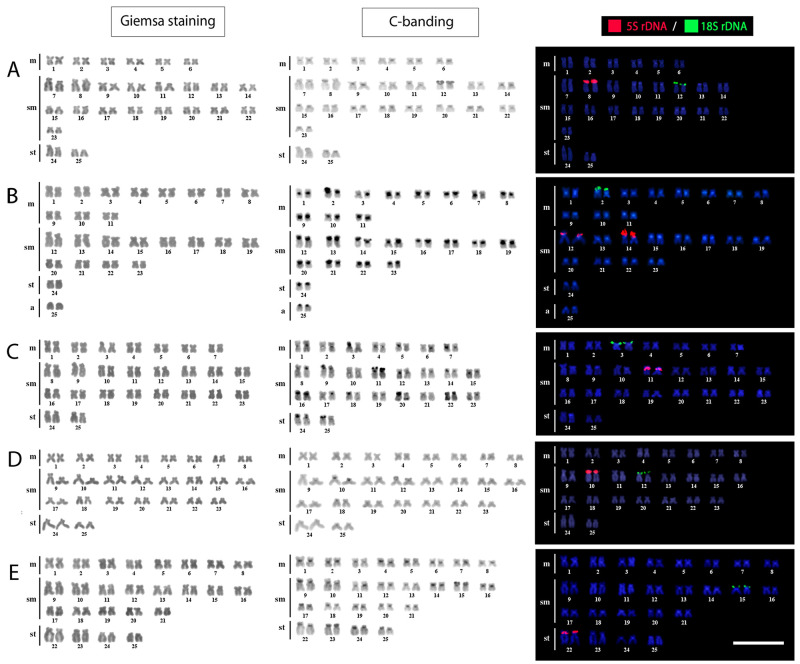
Karyotypes of the *Osteochilus* species examined arranged from Giemsa- stained, C-banded chromosomes and chromosomes after FISH with 5S (red) and 18S (green) rDNA probes. A= *O. lini*; B=*O. melanopleura*; C= *O. microcephalus*; D= *O. vittatus;* E= *O. waandersi*. Chromosomes were counterstained with DAPI (blue). Scale bar = 5 μm.

All five examined species possessed invariably, for both females and males, 2n = 50, but a different composition of their karyotypes: 12m+34sm+4st in *Osteochilus lini*, 22m+24sm+2st+2a in *O. melanopleura*, 14m+32sm+4st in *O. microcephalus*, 16m+30sm+4st in *O. vittatus* and 16m+26sm+8st in *O. waandersi* (Figure 2). The constitutive heterochromatin was always located at the pericentromeric region of all chromosomes. Additionally, the short (p) arms of some pairs also contained heterochromatic blocks, i.e., the 12^th^ in the karyotype of *O. lini*, 14^th^ of *O. melanopleura,* 11^th^ of *O. microcephalus,* 12^th^ of *O. vittatus* and the 15^th^ of *O. waandersi* (Figure 2).

FISH experiments documented a single pair bearing 5S and 18S rDNA sites in karyotypes of *Osteochilus lini* (pairs Nos. 08 and 12 respectively), *O. microcephalus* (Nos. 11 and 03), *O. vittatus* (Nos. 10 and 12) and in *O. waandersi* (Nos. 22 and 15), while in that of *O. melanopleura* 5S rDNA signals were situated on two chromosome pairs (Nos. 12 and 14) and only one pair with the 18S rDNA signal (No 02) (Figure 2).

**Figure 3 -:**
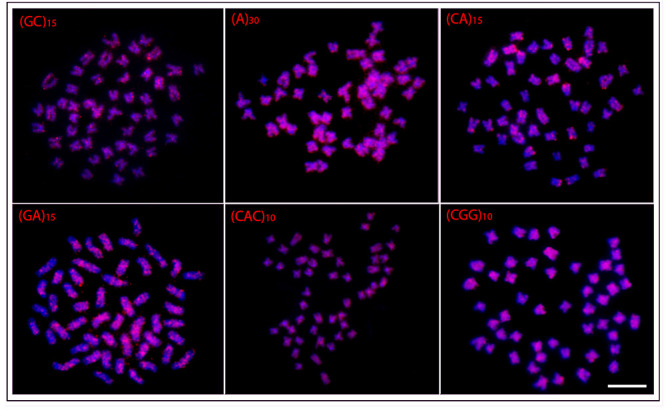
Hybridization patterns with microsatellites probes (red signals) on metaphase plates of *Osteochilus lini.* Chromosomes were counterstained with DAPI (blue). Scale bar = 5 μm.

**Figure 4 -:**
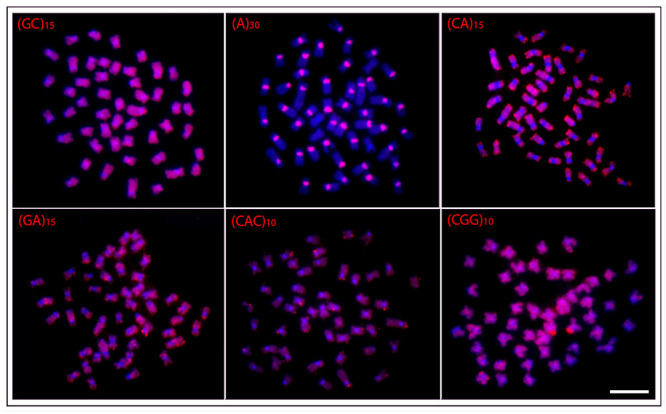
Hybridization patterns with microsatellites probes (red signals) on metaphase plates of the *Osteochilus melanopleura.* Chromosomes were counterstained with DAPI (blue). Scale bar = 5 μm.

**Figure 5 -:**
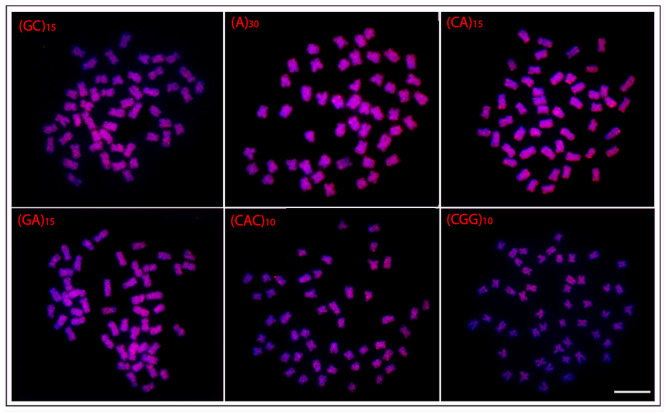
Hybridization patterns with microsatellites probes (red signals) on metaphase plates of the *Osteochilus microcephalus.* Chromosomes were counterstained with DAPI (blue). Scale bar = 5 μm.

**Figure 6 -:**
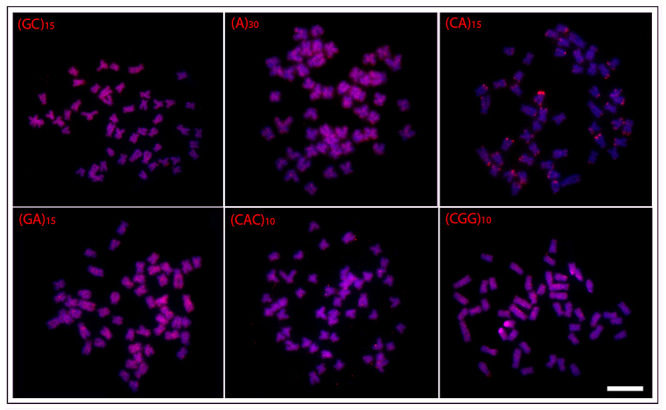
Hybridization patterns with microsatellites probes (red signals) on metaphase plates of the *Osteochilus vittatus.* Chromosomes were counterstained with DAPI (blue). Scale bar = 5 μm.

**Figure 7 -:**
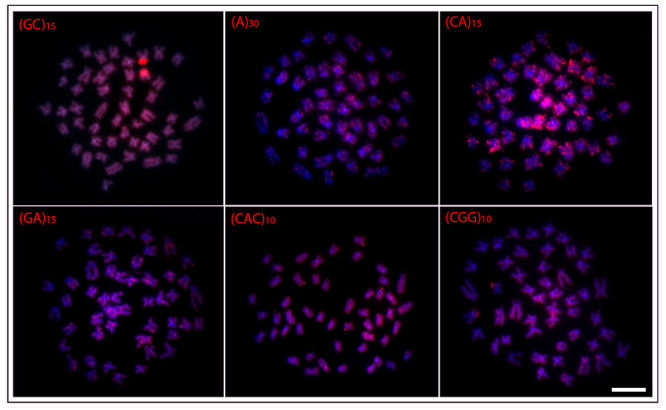
Hybridization patterns with microsatellites probes (red signals) on metaphase plates of the *Osteochilus waandersii.* Chromosomes were counterstained with DAPI (blue). Scale bar = 5 μm.

In general, a spreading pattern was a frequent feature for the microsatellites analyzed. However, some specific features could also be highlighted among species (Figures 3- 7). In this sense, *O. waandersi* had small spread (GC)_n_ signals in all chromosomes but a strong hybridization pattern in the pericentromeric region of a single pair. For (A)_30_, *O. melanopleura* showed the pericentromeric region of 46 chromosomes hybridized, while all the other species had scattered signals in all 50 chromosomes. Concerning (CA)_n_, while *O. microcephalus* and *O. waandersi* had a scattered distribution in all chromosomes, *O. lini* and *O. vittatus* presented small telomeric signals and *O. melanopleura* had scattered signals except in the centromeric regions. Spreading signals were also observed for the (GA)_n_, (CAC)_n_, and (CGG)_n_ probes in all chromosomes of all species. Additionally, *O. melanopleura* and *O. vittatus* had a strong (CGG)_n_ signal in the telomeric region of a single chromosome pair.

## Discussion

**Figure 8 -:**
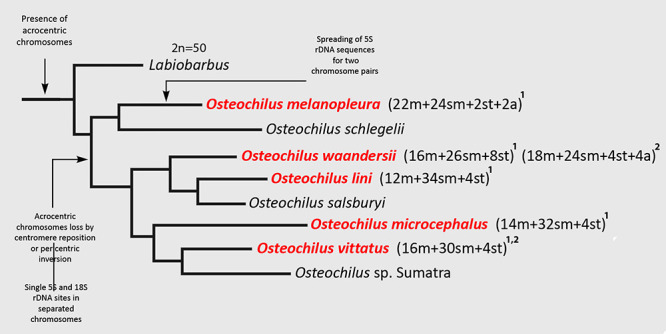
Adapted phylogenetic tree for the tribe Labeonini, based on the molecular-phylogenetic data generated by Yang et al. (2012) indicating the main chromosomal data obtained in this paper with the superscript 1, and by Magtoon and Arai (1990, 1993) with the superscript 2.

“Osteochilini” species possess 2n = 50 (Arai, 2011), which is also considered a basal pattern for cypriniform fishes (Chaiyasan *et al.*, 2018). Our results showed that 2n = 50 is also a demonstrably conserved pattern for all *Osteochilus* species karyotyped to date. However, despite the conservative 2n, significant differences in the karyotype structures in all five species examined were observed. Hence, this species also had multiple 5S rDNA sites and a different hybridization pattern for (A)_30_, (CA)_15_ and (CGG)_10_ microsatellites. According to the phylogeny of the Labeonini tribe proposed by Yang and Mayden (2010), *Osteochilus* was recovered as a monophyletic genus, with three *Labiobarbus* species forming a sister basal clade (Figure 8). *O. melanopleura* was recognized as the oldest derived species of the genus and *Labiobarbus lineatus* possessed 20 acrocentric chromosomes composing its karyotype (Magtoon and Arai, 1990). This fact suggests that the acrocentric pair No. 25 of *O. melanopleura* could be a remnant of the common ancestor between both *Osteochilus* and *Labiobarbus* genera. Thus, the karyotype diversification in *Osteochilus* genus was probably accompanied by a series of structural chromosome rearrangements, with a special role of pericentric inversions or centromere reposition, as indicated by changes in karyotype structure and a constant 2n (Figure 8).

Many representatives of several fish orders, such as Characiformes, Cypriniformes, Siluriformes, and Gymnotiformes have karyotypes dominated by bi-armed chromosomes (Molina *et al.*, 2014). Our data also demonstrated that *Osteochilus* species have more bi-armed elements in their karyotypes, suggesting that orthoselection and meiotic drift (White, 1973; Molina *et al.*, 2014) could be strong evolutionary drivers for this group. Noteworthy, the karyotype now reported for *O. waandersi* was different from that reported by Magtoon and Arai (1993).Cypriniform chromosomes have notable small sizes (Sember *et al.*, 2015; Saenjundaeng *et al.*, 2018) and this feature can make it difficult to visualize the correct centromere position (Ráb and Collares-Pereira, 1995; Spoz *et al.*, 2014; Knytl *et al.*, 2018), thus impairing the identification of the chromosomal morphology.

Microsatellite motifs had a preferential accumulation in heterochromatic regions (reviewed in Cioffi and Bertollo, 2012). However, the majority of the microsatellite sequences in *Osteochilus* showed a scattered pattern on chromosomes, without a specific relation with heterochromatic regions. Nevertheless, the (A)_30_ motif presented a strong accumulation pattern in the pericentromeric regions of *O. melanopleura*, a species in which this same chromosomal region appeared strongly C-banded, i.e., with C-positive heterochromatin. Also, microsatellites are often embedded within rDNA clusters (Piscor and Parise-Maltempi, 2016), which can also explain the strong labeling in the (CGG)_n_ motifs found in chromosomes of *O. vittatus* and *O. melanopleura*.

Usually, the 18S rDNA occupies a terminal position in chromosomes, in contrast to the more frequent interstitial position of the 5S rDNA (Sochorová *et al.*, 2018). All the *Osteochilus* species under study had both ribosomal classes located in a terminal position in association with heterochromatin, suggesting that these regions were recombination hotspots (Salvadori *et al.*, 1997; Sola *et al.*, 2003; Gornung, 2013). Their terminal position may also facilitate the dispersion of these sequences to other chromosomes, according to Rabl’s model, since higher recombination rates were found near the telomeric region (reviewed in Foster and Bridger, 2005). Besides that, the heterochromatinization of ribosomal loci was suggested to facilitate chromosomal heteromorphisms, by unequal crossing over between homologs and/or amplification of the heterochromatin between sister chromatids (Collares-Pereira and Ráb, 1999; Sola and Gornung, 2001; Gromicho *et al.*, 2006). The presence of both rDNAs in different chromosomal pairs is a usual condition in fish species (Sochorová *et al.*, 2018), as also observable for cyprinids in our study. Besides, it is noteworthy that *O. melanopleura* was recognized as a basal one in the genus (Karnasuta, 1993; Yang and Mayden, 2010; Figure 8), and this species had two chromosome pairs with 5S rDNA sites. In this sense, this could suggest that a single pair bearing such sites in the karyotypes of other *Osteochilus* species could be a derived pattern*.* However, this second pair with 5S sites in *O. melanopleura* was likely a particular pattern due to spreading events (Figure 8). Ribosomal clusters are characterized by its dynamism promoting significant intragenomic diversification (Gornung, 2013; Rebordinos *et al.*, 2013; Cioffi *et al.*, 2015; Sember *et al.*, 2015; Symonová and Howell, 2018). 

A general pattern on *Osteochilus* karyotypes with a fundamental number (NF) of 100 and a high variation on their karyotype macrostructure can generally be observed. This was somehow expected since *Osteochilus* is a specious genus, and it is known that the speciation process itself can be the result of high macrostructure karyotypic variation (White, 1973; Lowry and Willis, 2010). However, we cannot disregard the variation found in *O. melanopleura*, the variation that was also probably extended to the sister species *O. schlegelii*, but more studies are required to confirm this assumption. 

In conclusion, our data have improved the data about the karyotypes and chromosome characteristics in the genus *Osteochilus*. Its species presented a conservative 2n = 50 and NF = 100, but with differentiation of their karyotypes. Altogether these features indicate that chromosomal rearrangements, particularly the structural ones as centromere reposition and pericentric inversions, have taken place a major role during the evolutionary history of this cyprinid genus. The detailed cytogenetic survey indicated that the cytogenomic divergence patterns of these *Osteochilus* species were largely corresponding to the inferred phylogenetic tree. Also, repetitive DNAs, such as ribosomal and microsatellite ones, showed specificities in their distribution among species, thus being shown as good markers and promoters of specific genomic differentiation inside the genus.
